# Supplementary material for: Effects of Pressurized Aeration on the Biodegradation of Short-Chain Chlorinated Paraffins by Escherichia coli Strain 2
Source: Membranes (Basel). 2022 Jun 19;12(6):634. doi: 10.3390/membranes12060634 (PMC9227625; doi:10.3390/membranes12060634)
Supplement: Supplementary file 1 [file membranes-12-00634-s001.zip › membranes-1744516-supplementary.pdf]

Supplementary Material

Effects of pressurized aeration on the biodegradation of short-chain chlorinated paraffins by *Escherichia coli* strain 2

Yongxing Qian[1,2], Wanling Han[1,2], Fuhai Zhou[3], Bixiao Ji[1,2] and Huining Zhang[1,2]*and Kefeng Zhang[1,2]

[1] NingboTech University, Ningbo 315000, China; qianyx312@ nbt.edu.cn; hanwanling0325@outlook.com; jibixiao@nbt.edu.cn; kfzhang@nit.zju.edu.cn.
[2] Ningbo Research Institute, Zhejiang University, Ningbo 315100, China
[3] Zhejiang Haiyi Environmental Protection Equipment Engineering Co. Ltd. , Quzhou 324000,China ; zjhbfw@126.com.
* Correspondence: zhanghn@nbt.edu.cn; Tel./Fax: +86-15058033683

1. Dissolved oxygen (DO) concentration determination

This work used the Tromans thermodynamic equation (Eq. [1]) to describe the initial dissolved oxygen (DO) concentration inside the reactor under different pressures. Eq. (1) is applicable at temperatures from 273 K to 616 K and a fraction not exceeding 60 atm.

$$C_{aq}=P_{O_2}\exp\left\{\frac{0.046T^2+203.357T\ln\left(\frac{T}{298}\right)-(299.378+0.092T)(T-298)-20.591\times10^3}{8.3144T}\right\} \quad (1)$$

Table S1. Dissolved oxygen concentration at each pressure in the reactor.

Pressure (MPa)	DO concentration	
	Calculated value (mg/L)	Measured value (mg/L)
0.05	18.10	-
0.10	36.20	22.8
0.15	54.31	-
0.20	72.41	24.55
0.25	90.51	-
0.30	108.61	39.6

Note: "-" in Table 1 indicates no detection.

In this study, the reaction temperature was 33º℃(306.15 K), and the DO concentration at each pressure in the reactor was calculated. As shown in Table 1, the calculated DO value increased when the pressure increased. However, there was an enormous difference between the measured and calculated values. A possible reason was that the accuracy of the DO instrument decreased under pressure, and a higher pressure caused a greater decrease in accuracy. However, the measured value of DO also increased gradually upon increasing the pressure, and this trend was the same as the calculated value. In addition, our DO value was nearly three times higher than that determined by Xu et al.,[1] who reported a DO value of 12 mg/L at 0.3 MPa air pressure. This meant that at the same pressure, pure oxygen produced much more DO than air.

2. CSH determination

The improved bath method reported by Rosenberg et al.[2] was used for determination, and the CSH value was calculated using Eq. (2).

$$CSH(\%)=\left(1-\frac{OD_{600}}{OD_{600'}}\right)\times100\% \quad (2)$$

where OD_{600} is the OD_{600} value of the initial bacterial solution, and $OD_{600'}$ is the OD_{600} value of the final bacterial solution.

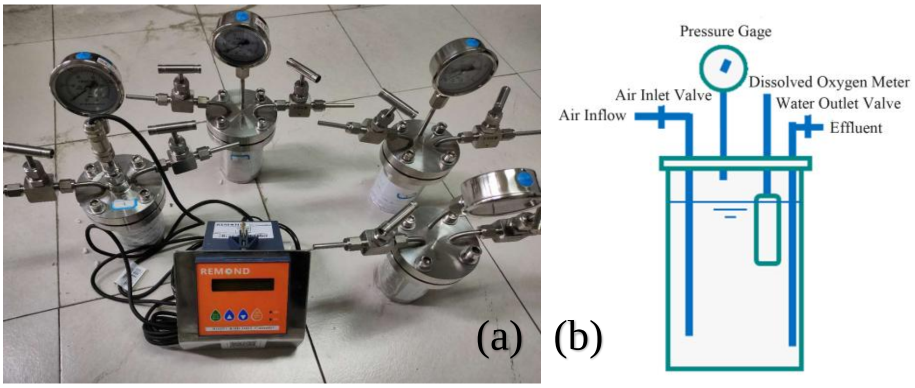

Figure S1. Pressurized bioreactor: (a) installation diagram; (b) schematic diagram.

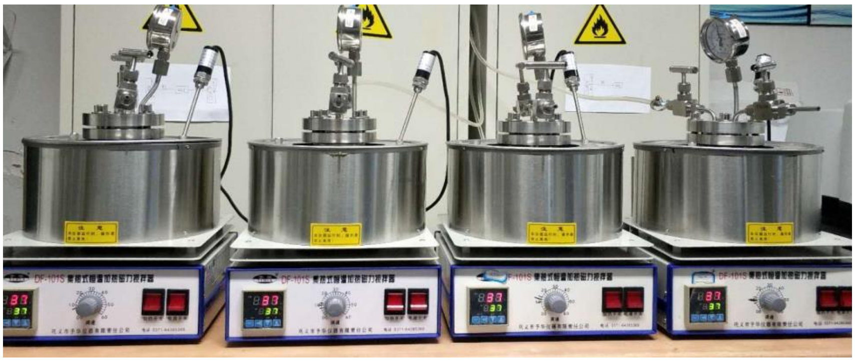

Figure S2. Pressurized bioreactor devices (constant temperature reaction at 33°C).

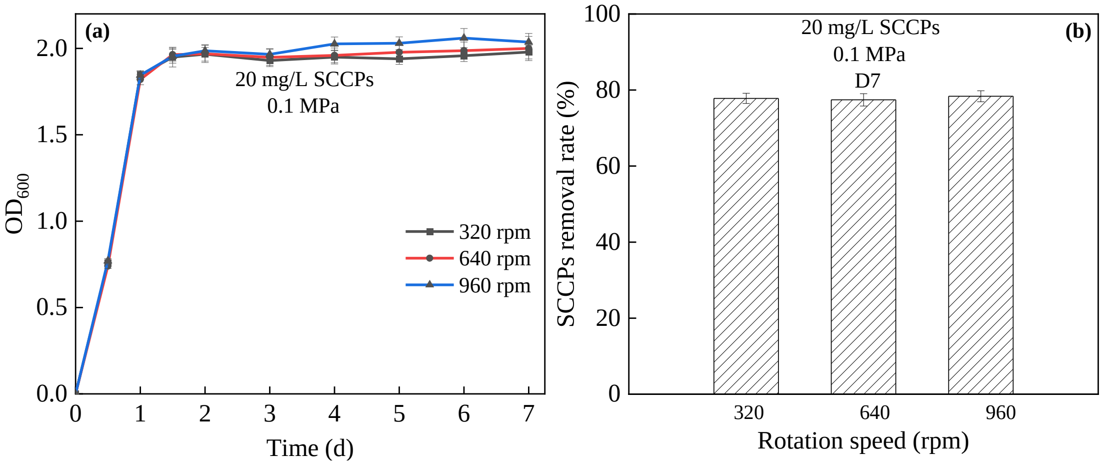

Figure S3. The relationship between different rotation speeds and (a) the growth of *E. coli* strain 2; (b) SCCPs removal rate in a pressurized reactor.

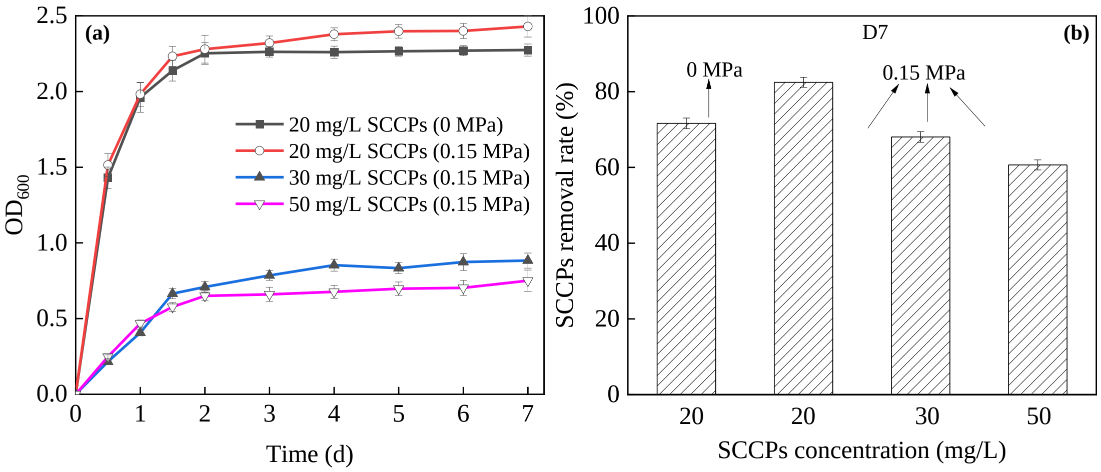

Figure S4. The relationship between the concentration of SCCPs and (a) the growth of *E. coli* strain 2; (b) SCCPs removal rate in a pressurized reactor.

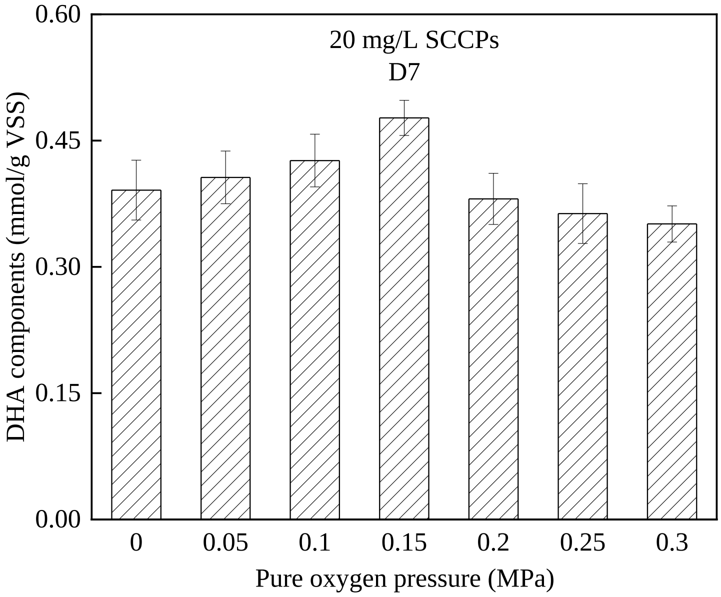

Figure S5. The relationship between different high-purity oxygen pressures and DHA in a pressurized reactor.

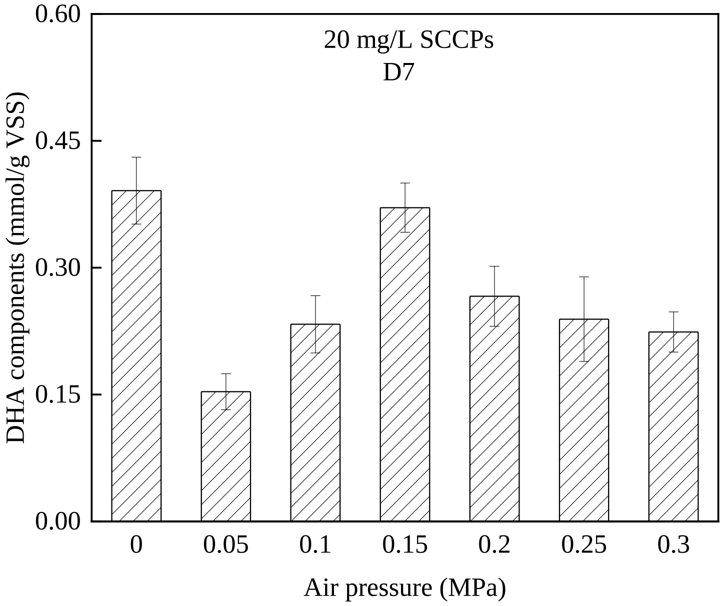

Figure S6. The relationship between different air pressures and DHA in a pressurized reactor.

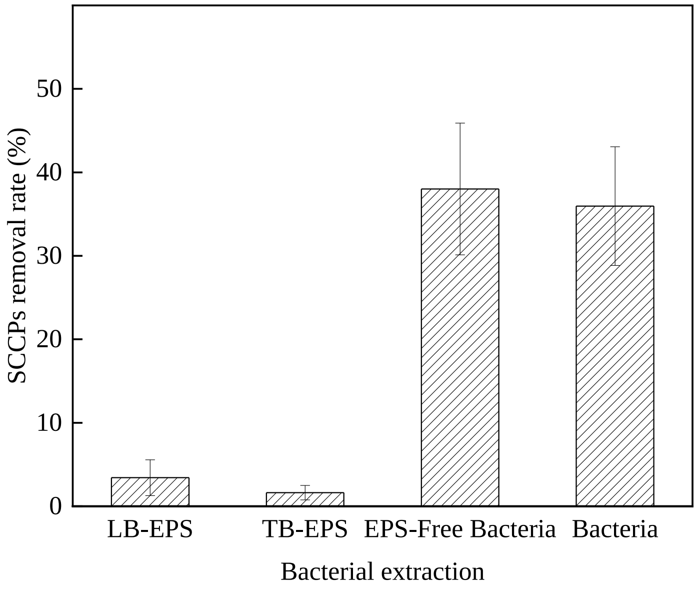

Figure S7. The adsorption of SCCPs by the extraction of *E. coli* strain 2.

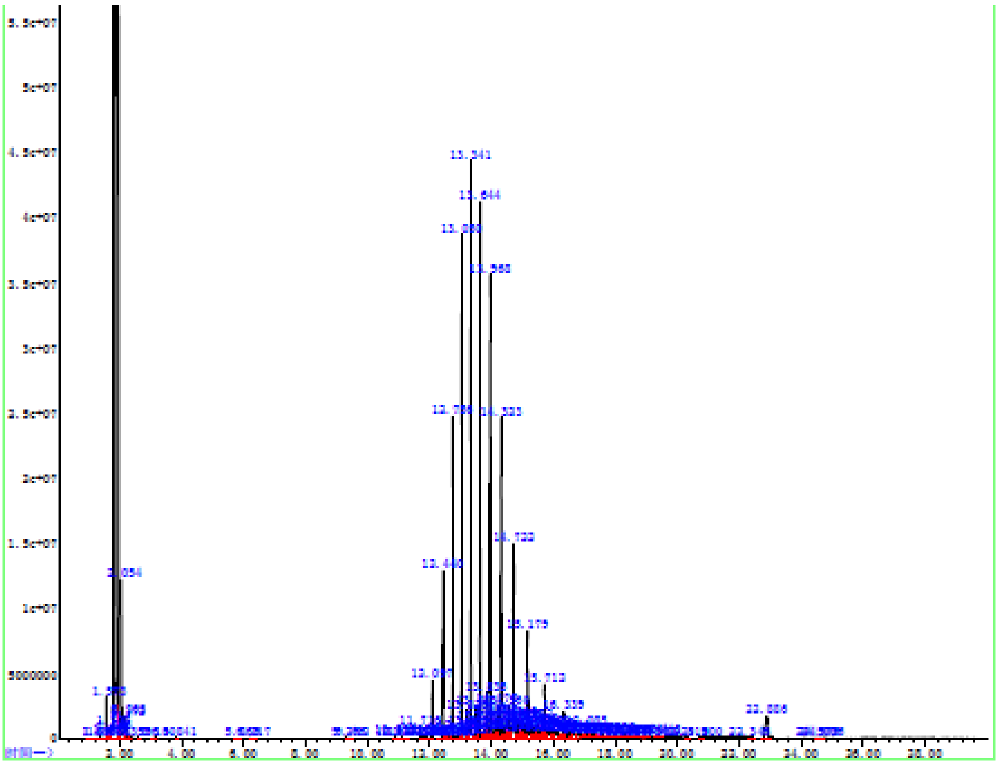

Figure S8. The GC-MS image of *E. coli* strain 2 degradation of SCCPs.

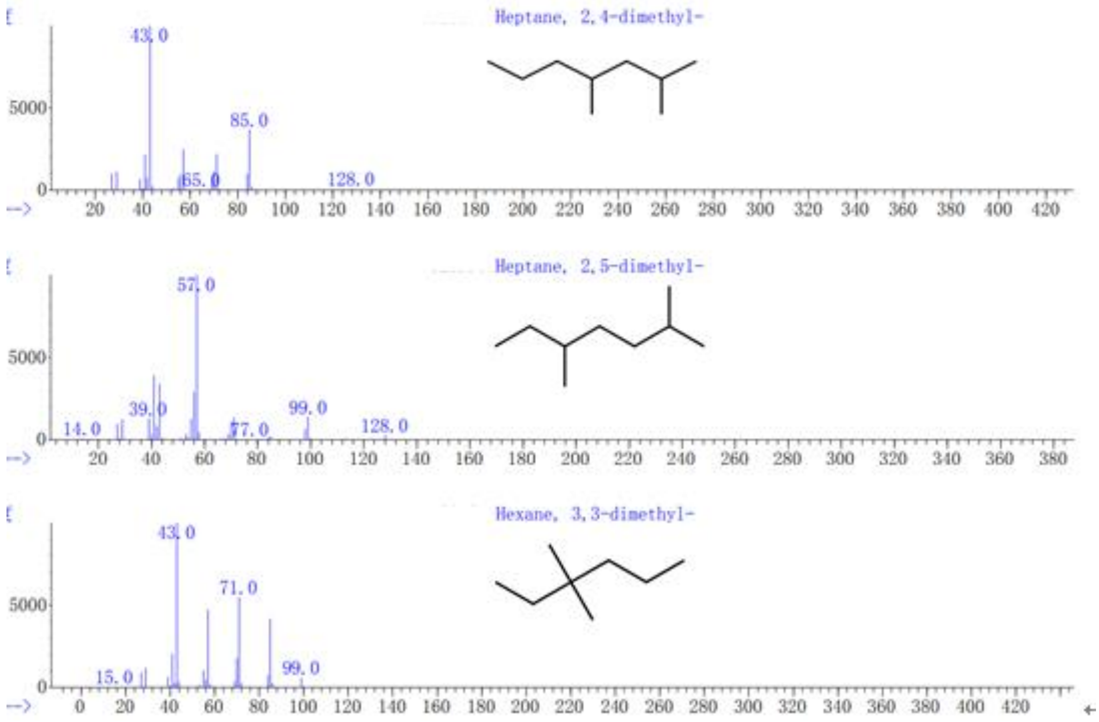

Figure S9. GC-MS spectra of the degradation products of SCCPs removed by *E. coli* strain 2.

Reference

[1] Xu, R.X.; Li, B.; Zhang, Y.; Si, L.; Zhang, X. Q.; Xie, B. Response of biodegradation characteristics of unacclimated activated sludge to moderate pressure in a batch reactor. *Chemosphere* **2016**,148, 41–46.
[2] Rosenberg, M.; Gutnick, D.; Rosenberg, E. Adherence of bacteria to hydrocarbons: A simple method for

measuring cell-surface hydrophobicity. *FEMS Microbiol. Lett.* **1980**, 9(1), 29–33.
